# Supplementary material for: Shc1 cooperates with Frs2 and Shp2 to recruit Grb2 in FGF-induced lens development
Source: bioRxiv. 2025 Feb 17:2024.10.20.619055. Originally published 2024 Oct 22. Preprint. [Version 3] doi: 10.1101/2024.10.20.619055 (PMC11527007; doi:10.1101/2024.10.20.619055)
Supplement: Supplement 1 — Supplementary Figure 1. Lens development in Fgf receptor mutants. (A) Depletion of all four Fgfr1/2/3/4 did not disrupt the Fibronectin expression at the basal side of the lens placode (arrows). (B) pmTOR and pS6 staining were reduced in Le-Cre;Fgfr1f/f;Fgfr2f/LR lens. (C) Quantification of pmTOR fluorescent intensity in Fgfr1/2 mutants. Student’s t-test, n=3, P<0.0001. (D) Quantification of pmS6 fluorescent intensity in Fgfr1/2 mutants. Student’s t-test, n=3, P<0.01. Supplementary Figure 2. Cell proliferation and apoptosis in Shp2CS mutants. Le-Cre;Shp2f/CS lens exhibits normal expression of proliferation marker Ki67, but there is a significant increase in TUNLE+ cells (arrowheads). Student’s t-test, N.S. (not significant) for %Ki67+ cells and P<0.01 for %TUNEL+ cells in the lens epithelium. [file NIHPP2024.10.20.619055v3-supplement-1.pdf]

bioRxiv preprint doi: https://doi.org/10.1101/2024.10.20.619055; this version posted February 17, 2025. The copyright holder for this preprint (which was not certified by peer review) is the author/funder, who has granted bioRxiv a license to display the preprint in perpetuity. It is made available under aCC-BY 4.0 International license.

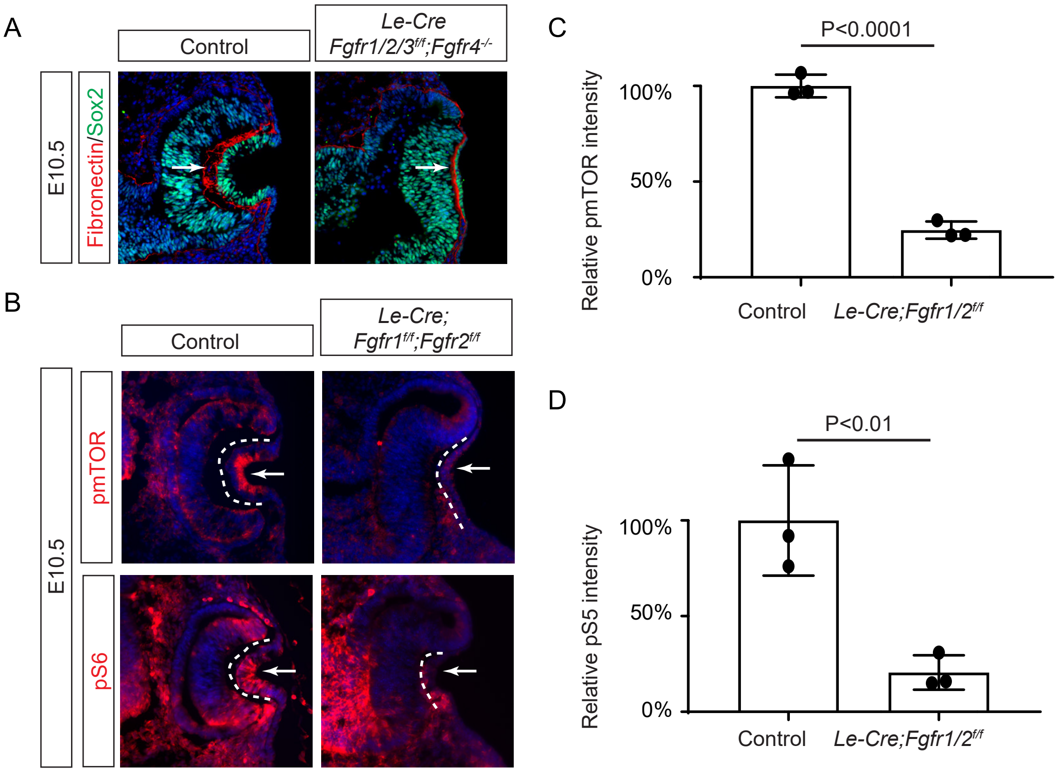

Supplementary Figure 1. Lens development in Fgf receptor mutants. (A) Depletion of all four *Fgfr1/2/3/4* did not disrupt the Fibronectin expression at the basal side of the lens placode (arrows). **(B)** pmTOR and pS6 staining were reduced in *Le-Cre;Fgfr1$^{f/f}$;Fgfr2$^{f/LR}$* lens. **(C)** Quantification of pmTOR fluorescent intensity in *Fgfr1/2* mutants. Student's t-test, n=3, P<0.0001. **(D)** Quantification of pmS6 fluorescent intensity in *Fgfr1/2* mutants. Student's t-test, n=3, P<0.01.

bioRxiv preprint doi: https://doi.org/10.1101/2024.10.20.619055; this version posted February 17, 2025. The copyright holder for this preprint (which was not certified by peer review) is the author/funder, who has granted bioRxiv a license to display the preprint in perpetuity. It is made available under aCC-BY 4.0 International license.

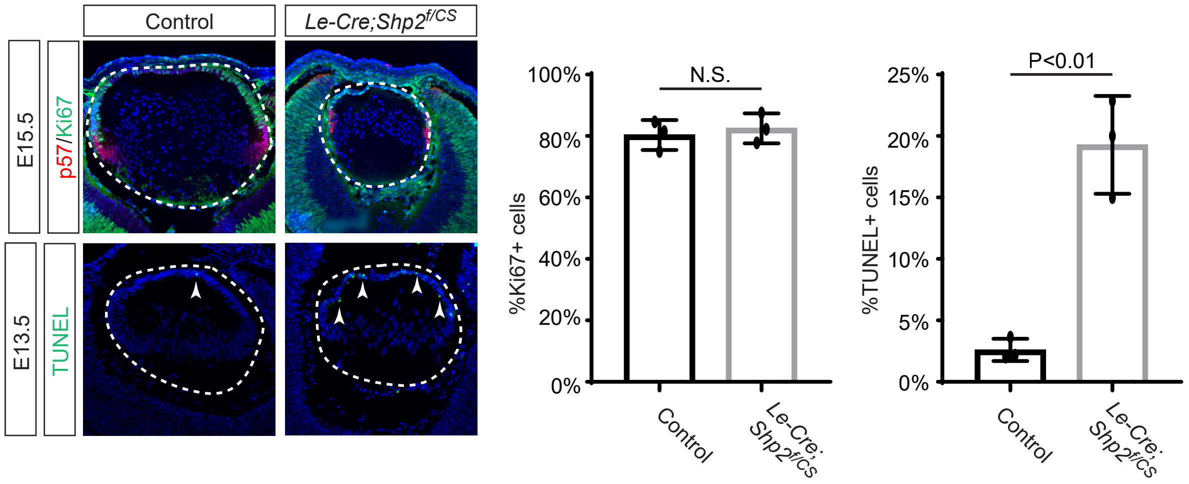

Supplementary Figure 2. Cell proliferation and apoptosis in ***Shp2^{CS}*** **mutants.** *Le-Cre;Shp2$^{f/CS}$* lens exhibits normal expression of proliferation marker Ki67, but there is a significant increase in TUNLE+ cells (arrowheads). Student's t-test, N.S. (not significant) for %Ki67+ cells and P<0.01 for %TUNEL+ cells in the lens epithelium.
