# Supplementary material for: Shc1 cooperates with Frs2 and Shp2 to recruit Grb2 in FGF-induced lens development
Source: bioRxiv. 2024 Oct 22:2024.10.20.619055. Preprint. [Version 1] doi: 10.1101/2024.10.20.619055 (PMC11527007; doi:10.1101/2024.10.20.619055)
Supplement: Supplementary file 1 [file NIHPP2024.10.20.619055V1-supplement-1.pdf]

bioRxiv preprint doi: https://doi.org/10.1101/2024.10.20.619055; this version posted October 22, 2024. The copyright holder for this preprint (which was not certified by peer review) is the author/funder, who has granted bioRxiv a license to display the preprint in perpetuity. It is made available under aCC-BY 4.0 International license.

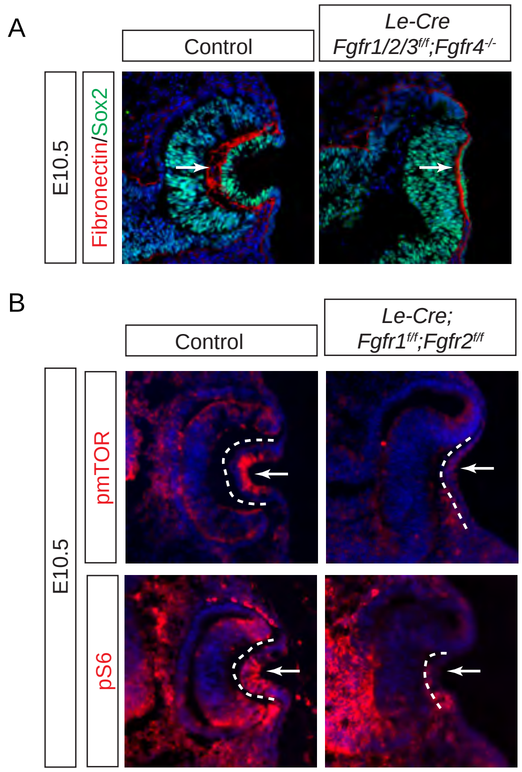

Supplementary Figure 1. Lens development in Fgf receptor mutants. (A) Depletion of all four *Fgfr1/2/3/4* did not disrupt the Fibronectin expression at the basal side of the lens placode (arrows). **(B)** pmTOR and pS6 staining were lost in *Le-Cre;Fgfr1*$^{f/f}$*;Fgfr2*$^{f/LR}$ lens.

bioRxiv preprint doi: https://doi.org/10.1101/2024.10.20.619055; this version posted October 22, 2024. The copyright holder for this preprint (which was not certified by peer review) is the author/funder, who has granted bioRxiv a license to display the preprint in perpetuity. It is made available under aCC-BY 4.0 International license.

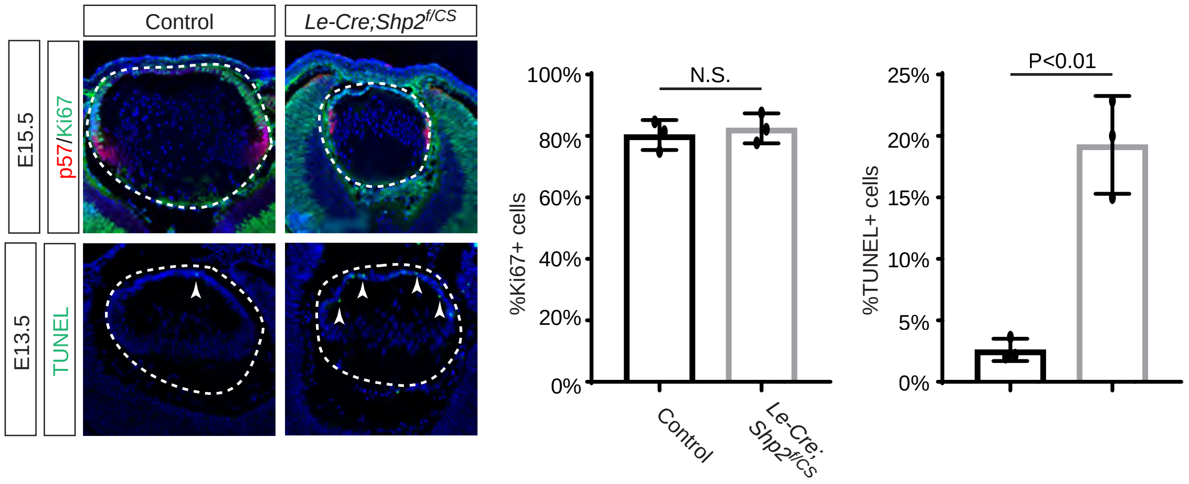

Supplementary Figure 2. Cell proliferation and apoptosis in ***Shp2***^{CS} **mutants.** *Le-Cre;Shp2*$^{f/CS}$ lens exhibits normal expression of proliferation marker Ki67, but there is a significant increase in TUNLE+ cells (arrowheads). Student's t-test, N.S. (not significant) for %Ki67+ cells and P<0.01 for %TUNEL+ cells in the lens epithelium.
